# Identification and validation of endoplasmic reticulum stress-related diagnostic biomarkers for type 1 diabetic cardiomyopathy based on bioinformatics and machine learning

**DOI:** 10.3389/fendo.2025.1478139

**Published:** 2025-03-18

**Authors:** Qiao Tang, Yanwei Ji, Zhongyuan Xia, Yuxi Zhang, Chong Dong, Qian Sun, Shaoqing Lei

**Affiliations:** ^1^ Department of Anesthesiology, Renmin Hospital of Wuhan University, Wuhan, China; ^2^ Organ Transplantation Center, Tianjin First Central Hospital, Tianjin, China; ^3^ Tianjin Key Laboratory for Organ Transplantation, Tianjin, China

**Keywords:** type 1 diabetes, diabetic cardiomyopathy, endoplasmic reticulum stress, immune infiltration, bioinformatics, marker genes

## Abstract

**Background:**

Diabetic cardiomyopathy (DC) is a serious complication in patients with type 1 diabetes mellitus and has become a growing public health problem worldwide. There is evidence that endoplasmic reticulum stress (ERS) is involved in the pathogenesis of DC, and related diagnostic markers have not been well-studied. Therefore, this study aimed to screen ERS-related genes (ERGs) with potential diagnostic value in DC.

**Methods:**

Gene expression data on DC were downloaded from the GEO database, and ERGs were obtained from The Gene Ontology knowledgebase. Limma package analyzed differentially expressed genes (DEGs) in the DC and control groups, and then integrated with ERGs to identify ERS-related DEGs (ERDEGs). The ERDEGs diagnostic model was developed based on a combination of LASSO and Random Forest approaches, and the diagnostic performance was evaluated by the area under the receiver operating characteristic curve (ROC-AUC) and validated against external datasets. In addition, the association of the signature genes with immune infiltration was analyzed using the CIBERSORT algorithm and the Spearman correlation test.

**Results:**

Gene expression data on DC were downloaded from the GEO database and ERGs were obtained from the Gene Ontology Knowledgebase. Limma package analysis identified 3100 DEGs between DC and control groups and then integrated with ERGs to identify 65 ERDEGs. Four diagnostic markers, Npm1, Jkamp, Get4, and Lpcat3, were obtained based on the combination of LASSO and random forest approach, and their ROC-AUCs were 0.9112, 0.9349, 0.8994, and 0.8639, respectively, which proved their diagnostic potential in DC. Meanwhile, Npm1, Jkamp, Get4, and Lpcat3 were validated by external datasets and a mouse model of type 1 DC. In addition, Npm1 was significantly negatively correlated with plasma cells, activated natural killer cells, or quiescent mast cells, whereas Get4 was significantly positively correlated with quiescent natural killer cells and significantly negatively correlated with activated natural killer cells (*P* < 0.05).

**Conclusions:**

This study provides novel diagnostic biomarkers (Npm1, Jkamp, Get4, and Lpcat3) for DC from the perspective of ERS, which provides new insights into the development of new targets for individualized treatment of type 1 diabetic cardiomyopathy.

## Introduction

Type 1 diabetes mellitus is a chronic metabolic disease that threatens global health, with the latest epidemiology showing that it accounts for about two percent of the nearly 500 million total diabetes mellitus worldwide and is rising for some unknown reason (1, 2). Persuasive studies show that cardiovascular complications are the dominant cause of morbidity and mortality in Type 1 diabetes mellitus (3, 4). Diabetic cardiomyopathy (DC) is defined as structural and functional abnormalities of the heart in the absence of coronary artery disease, hypertension, and valvular heart disease, manifested by diastolic and systolic dysfunction, and ultimately progressing to heart failure, arrhythmias, and even sudden death from cardiogenic shock (5, 6). Potential mechanisms include oxidative stress, inflammation, and calcium impairment, as well as alterations in substrate metabolism/utilization, insulin signaling, gene regulation, mitochondrial dysfunction, endoplasmic reticulum stress (ERS), neurohumoral activation, and cell death (7). Currently, there is no specific treatment for DC, and a large number of patients irreversibly progress to heart failure. Therefore, it is crucial to identify effective biomarkers for early diagnosis and treatment.

The endoplasmic reticulum (ER) of mammalian cells serves as the primary site for protein folding and assembly, establishing its role as “the core organelle that ensures normal cell function” (8). Under normal conditions, misfolded proteins in the body trigger an unfolded protein response (UPR) within the endoplasmic reticulum lumen, effectively mitigating the adverse effects of misfolded proteins and potentially preventing disease onset (8, 9). However, it is important to note that in the presence of metabolic diseases such as diabetes, ocular conditions like age-related macular degeneration and retinitis pigmentosa, or cardiovascular diseases, liver disease and even cancer, factors such as oxidative stress, metabolic abnormalities, and Ca^2+^ dysregulation become widely activated (8, 10–13). These factors can lead to a significant increase in unfolded and misfolded proteins, resulting in the overactivation of the unfolded protein response and consequently inducing endoplasmic reticulum stress (8, 14–17). Following the occurrence of endoplasmic reticulum stress, downstream pathways are primarily activated through three signaling proteins: inositol-requiring protein-1α (IRE1α), protein kinase RNA-like ER kinase (PERK), and activating transcription factor 6 (ATF6) (18–20). These pathways ultimately inhibit protein synthesis, regulate gene expression, and determine cell fate, including processes such as apoptosis. In summary, endoplasmic reticulum stress serves as a sensitive sensor for the onset of disease and plays a crucial role in determining the final fate of cells. A significant number of studies have indicated that diabetic cardiomyopathy, a serious condition characterized by various risk factors including oxidative stress, metabolic abnormalities, and Ca^2+^ overload, is closely associated with endoplasmic reticulum stress (21–23). Furthermore, many investigations have highlighted that endoplasmic reticulum-related genes may serve as early markers of ischemic heart disease and play a crucial role in its pathophysiology (24, 25). Building on this foundation, we propose the scientific research hypothesis that endoplasmic reticulum-related genes may be involved in the progression of diabetic cardiomyopathy and could potentially serve as predictive markers for identifying intervention targets.

Many recent studies have emphasized that immune responses and ERS crosstalk with each other and are fundamentally and comprehensively intertwined (26–28). The effects of ERS include direct defense against microbial pathogens, production of pro-inflammatory cytokines, presentation of antigens to T cells, immunogenic cell death, metabolic homeostasis, and maintenance of immune tolerance (29). Effective immunity depends on endoplasmic reticulum homeostatic processes such as calcium signaling, glycosylation, lipid metabolism, and oxidative protein folding (30). During transient ER stress, all three signaling pathways of the UPR can crosstalk with inflammatory and stress signaling pathways, including nuclear factor-kappa B (NF-κB), a major transcriptional regulator of innate immunity (31). In view of this, ERGs and transcriptomic data on type 1 DC were collected from public databases, and ERS-related differentially expressed genes (ERDEGs) between DC and control samples were identified, which screened potential ERS-related diagnostic markers, providing new ideas for the diagnosis and treatment of DC.

## Result

### Identification and functional enrichment analysis of differentially expressed genes

**Figure 1:**
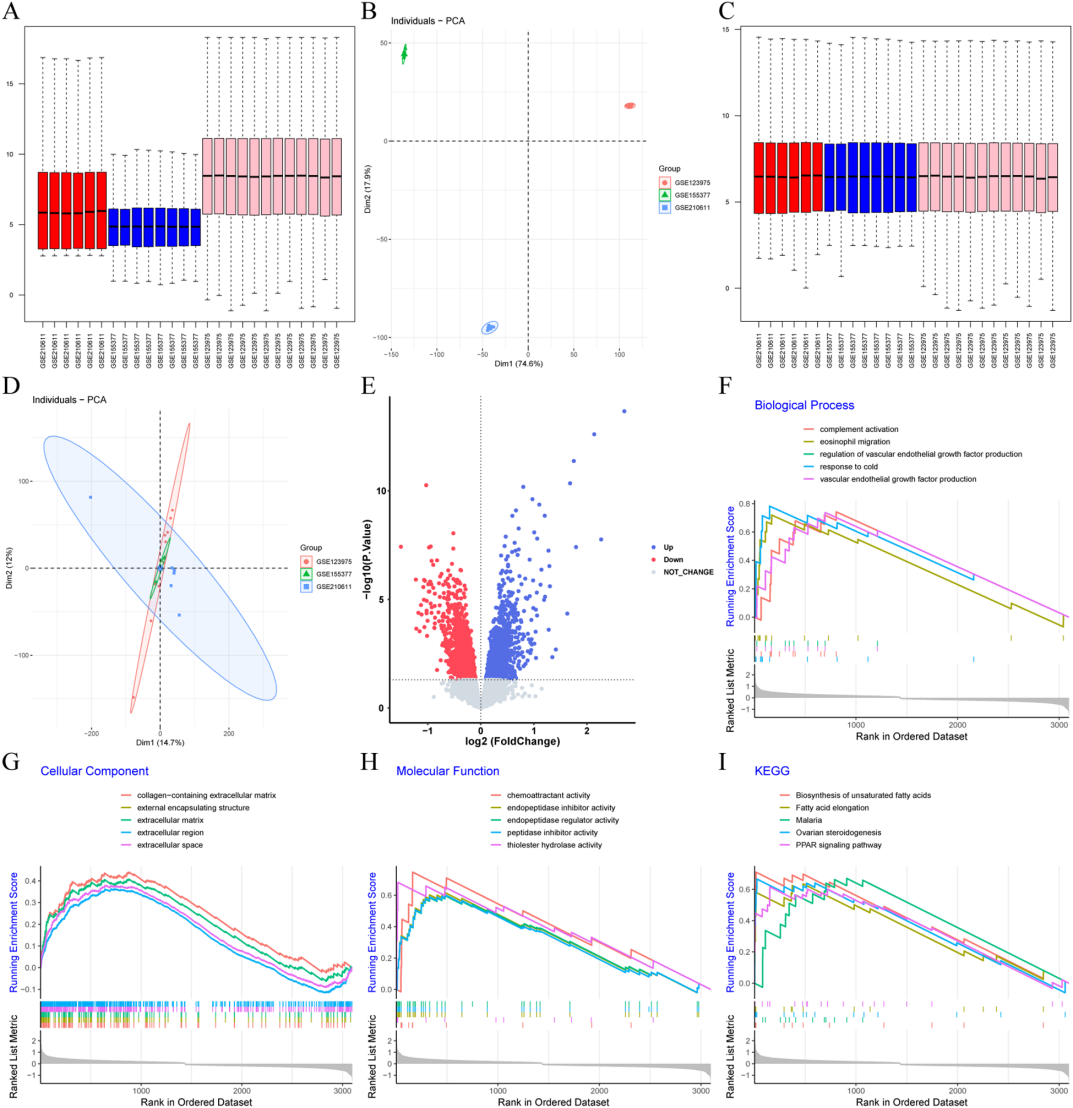
Identification and functional enrichment analysis of DEGs. **(A-D)** Boxplots and PCA were applied to visualize the batch correction effect before **(A, B)** and after **(C, D)** batch effect removal. **(E)** Volcano plot of DEGs. Blue and red dots indicate significantly up-and down-regulated genes, respectively, while gray dots indicate genes with no significant difference. **(F)** Top ten biological processes active in DC. **(G)** Top ten cellular components active in DC. **(H)** Top ten molecular functions active in DC. **(I)** Top ten KEGG pathways active in DC.

To increase the sample size for enhanced confidence and reliability of the results, GSE155377, GSE210611, and GSE123975 were combined into one cohort, which ultimately consisted of 13 control samples and 13 DC samples. Box plot analysis (**Figures 1A, C**) and PCA (**Figures 1B, D**) indicated that batch effects were successfully eliminated. The batch-corrected PCA analysis showed that the data distribution tended to be uniform across the datasets, implying that normalization might be completed correctly. A total of 3100 DEGs were identified, of which 1662 were down-regulated and 1438 were up-regulated (**Figure 1E**). In addition, gene set enrichment analysis revealed that the biological processes (BP) involved in DC mainly consisted of complement activation, eosinophil migration, regulation of vascular endothelial growth factor production, response to cold and vascular endothelial growth factor production (**Figure 1F**). Cellular components (CC) involved in DC mainly consisted of collagen-containing extracellular matrix、external encapsulating structure, extracellular matrix, extracellular region and extracellular space (**Figure 1G**). molecular functions (MF) involved in DC mainly consisted of chemoattractant activity, endopeptidase inhibitor activity, endopeptidase regulator activity, peptidase inhibitor activity and thioester hydrolase activity (**Figure 1H**). The active Kyoto Encyclopedia of Genes and Genomes (KEGG) pathways were mainly biosynthesis of unsaturated fatty acids, fatty acid elongation, malaria, ovarian steroidogenesis, and PPAR signaling pathways (**Figure 1I**).

### Identification and functional annotation analysis of endoplasmic reticulum stress-related differentially expressed genes

**Figure 2:**
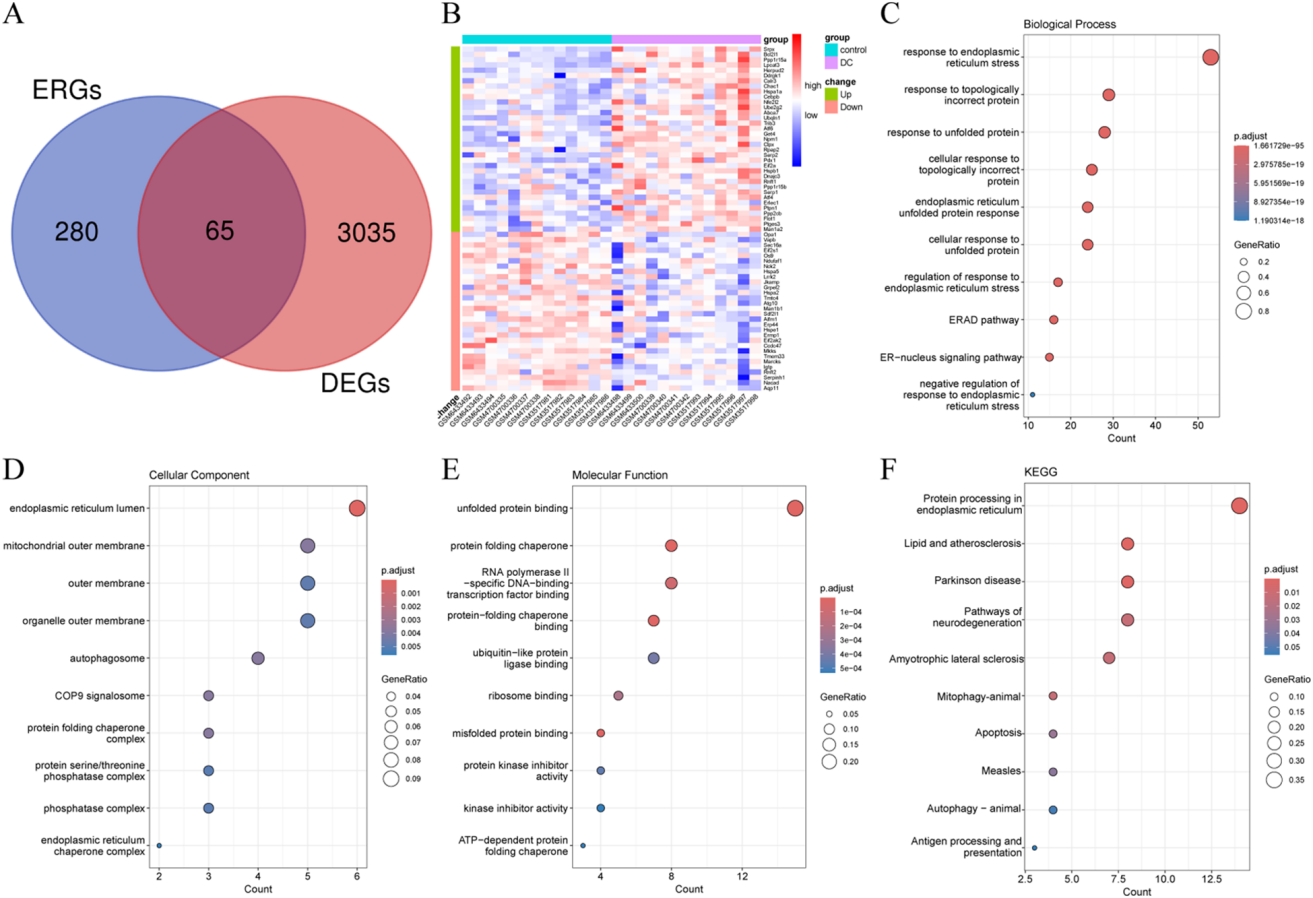
Identification and functional annotation analysis of ERDEGs. **(A)** Venn diagram illustrating the overlap region between DEGs and ERGs in DC. **(B)** Heatmap demonstrating the expression levels of ERDEGs in each sample. **(C-E)** Dot plots of the top 10 highest enrichment levels for biological processes, cellular components, and molecular functions. **(F)** Dot plots of the top 10 pathway terms with the highest enrichment levels identified by KEGG analysis.

Sixty-five ERDEGs were obtained by crossing 345 ERGs with DEGs (**Figure 2A**). Of these, 35 were up-regulated and 30 were down-regulated (**Figure 2B**). Functional annotation analysis was applied to further explore the functions of ERDEGs, and as a result, many terms related to ERS were enriched. The BP was mainly enriched for response to ERS, cellular response to topologically incorrect protein, and cellular response to unfolded protein (**Figure 2C**), suggesting that their perturbation may mediate the pathogenesis of DC. The CC was mainly enriched for the endoplasmic reticulum lumen, organelle outer membrane, protein folding chaperone complex, etc. (**Figure 2D**). The MF was significantly enriched for unfolded protein binding, protein-folding chaperone binding, and misfolded protein binding (**Figure 2E**). Of particular note, KEGG analysis revealed that the top-ranked pathway was protein processing in the endoplasmic reticulum (**Figure 2F**).

### Identification and ROC analysis of ERS-related signature genes by LASSO algorithm and RandomForest

**Figure 3:**
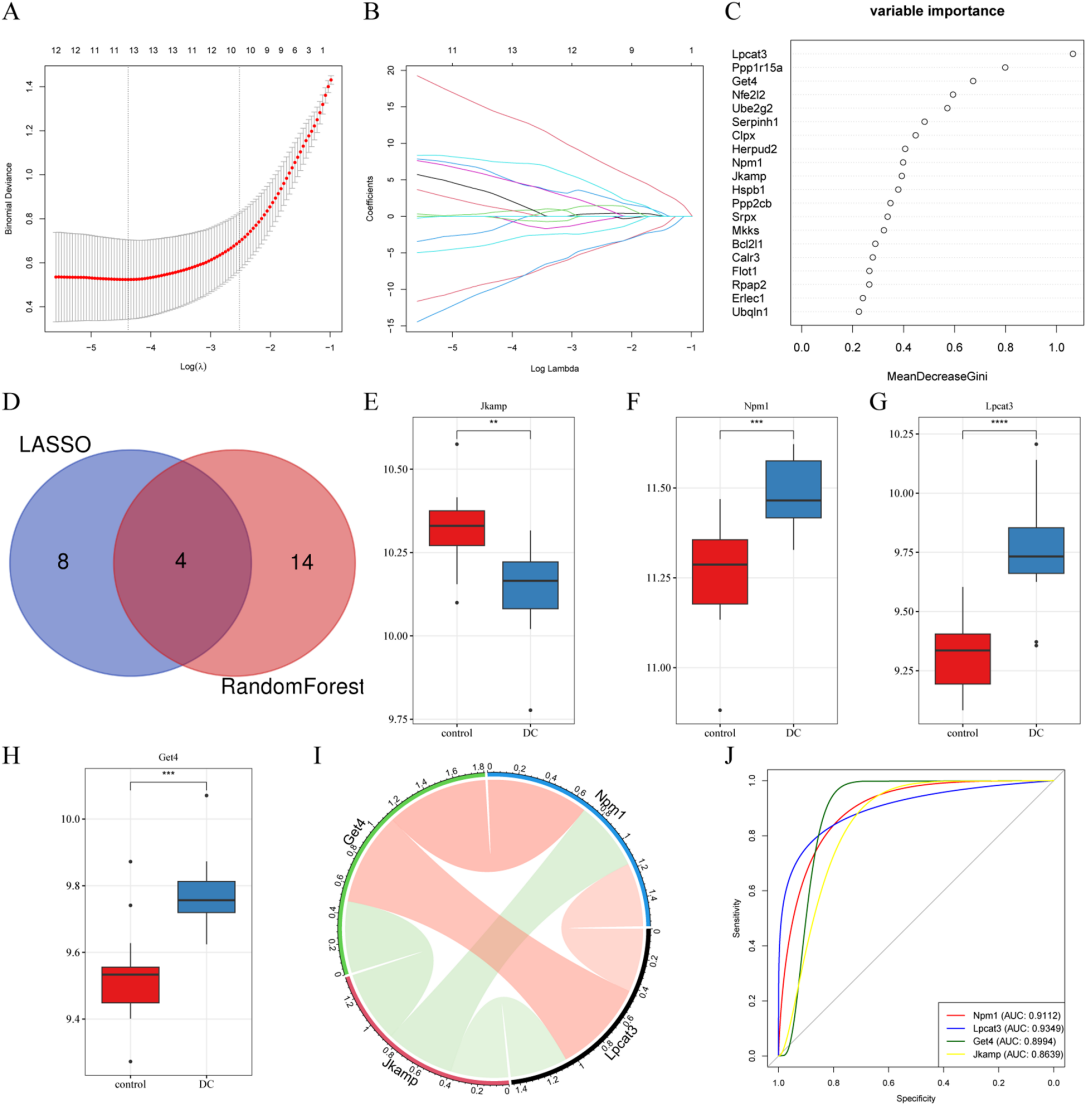
Selection of signature genes by machine learning algorithms. **(A)** Schematic diagram of the LASSO regression diagnostic model for ERDEGs. **(B)** Trajectory diagram describing the LASSO selection variables in the diagnostic model for ERDEGs. **(C)** Dotplot of relative importance ranking by RandomForest. **(D)** Venn diagram of common signature genes. **(E-H)** Boxplots depicting the expression levels of four signature genes in the constructed features. ^**^P < 0.01, ^***^P < 0.001, ^****^P < 0.0001. **(I)** Chord plot illustrating the correlation of the characterized genes. **(J)** ROC analysis of the four characterized genes.

When LASSO was constructed based on 10-fold cross-validation, the minimum error value corresponded to 12 signature genes, including Pik3r1, Tomm20, Prkn, Rasgrf2, Sgta, Scamp5, Rcn3, and Pdia3 (**Figures 3A, B**). Using 0.25 as the importance score threshold, 18 signature genes were obtained, including Npm1, Jkamp, Get4, Lpcat3, Ppp2cb, Mkks, Clpx, Bcl2l1, Ube2g2, Flot1, Ppp1r15a, Nfe2l2, Rpap2, Srpx, Calr3, Serpinh1, Hspb1, and Herpud2 (**Figure 3C**). Npm1, Jkamp, Get4, and Lpcat3 are signature genes common to LASSO and RandomForest (**Figure 3D**). Among them, Jkamp was down-regulated in DC (**Figure 3E**) and all other genes were up-regulated (**Figures 3F–H**). Significant positive correlations were observed between Lpcat3 or Get4 and Npm1, and between Lpcat3 and Get4, whereas significant negative correlations were observed between Jkamp and the other three genes (**Figure 3I**). ROC-AUCs of the four signature genes in the combined dataset were all greater than 0.75, indicating excellent diagnostic capability (**Figure 3J**).

### Correlation of signature genes with infiltrating immune cells

**Figure 4:**
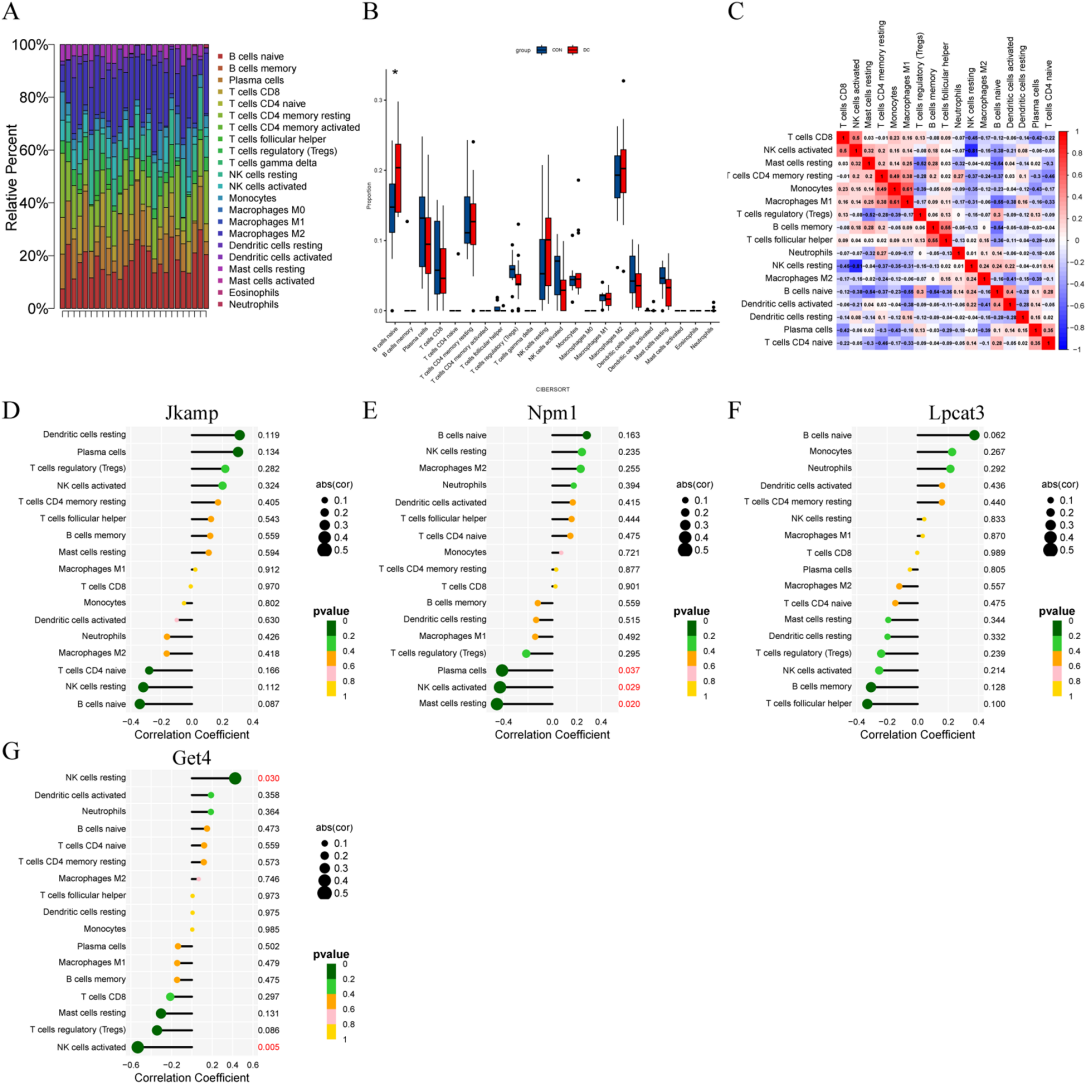
Correlation of signature genes with infiltrating immune cells. **(A)** Cumulative histograms of the distribution of 22 immune cells in the DC and control groups. **(B)** Box plots of immune cell expression in control and DC groups. ^*^P < 0.05. **(C)** Heatmap of correlation between immune cells. **(D-G)** Lollipop plots of correlation between immune cells and signature genes.

Cumulative histograms showed the relative proportions of 22 immune cells in DC and control samples, with B cells naive, Macrophages M2, T cells CD4 memory resting, and Plasma cells accounting for the majority (**Figure 4A**). Boxplots of differences in immune cell infiltration showed significantly more B cells naive in the DC group than in the control group (*P* < 0.05; **Figure 4B**). The heatmap displayed the correlation between the 22 immune cell types (**Figure 4C**). The correlation of Jkamp and Lpcat3 with 22 immune cell types was not statistically significant (**Figures 4D, F**). Npm1 showed a significant negative correlation with Plasma cells, natural killer cells activated, or Mast cells resting (**Figure 4E**). Get4 showed a significant positive correlation with natural killer cells resting and a significant negative correlation with natural killer cells activated (**Figure 4G**).

### Validation of the expression and diagnostic ability of the signature genes

**Figure 5:**
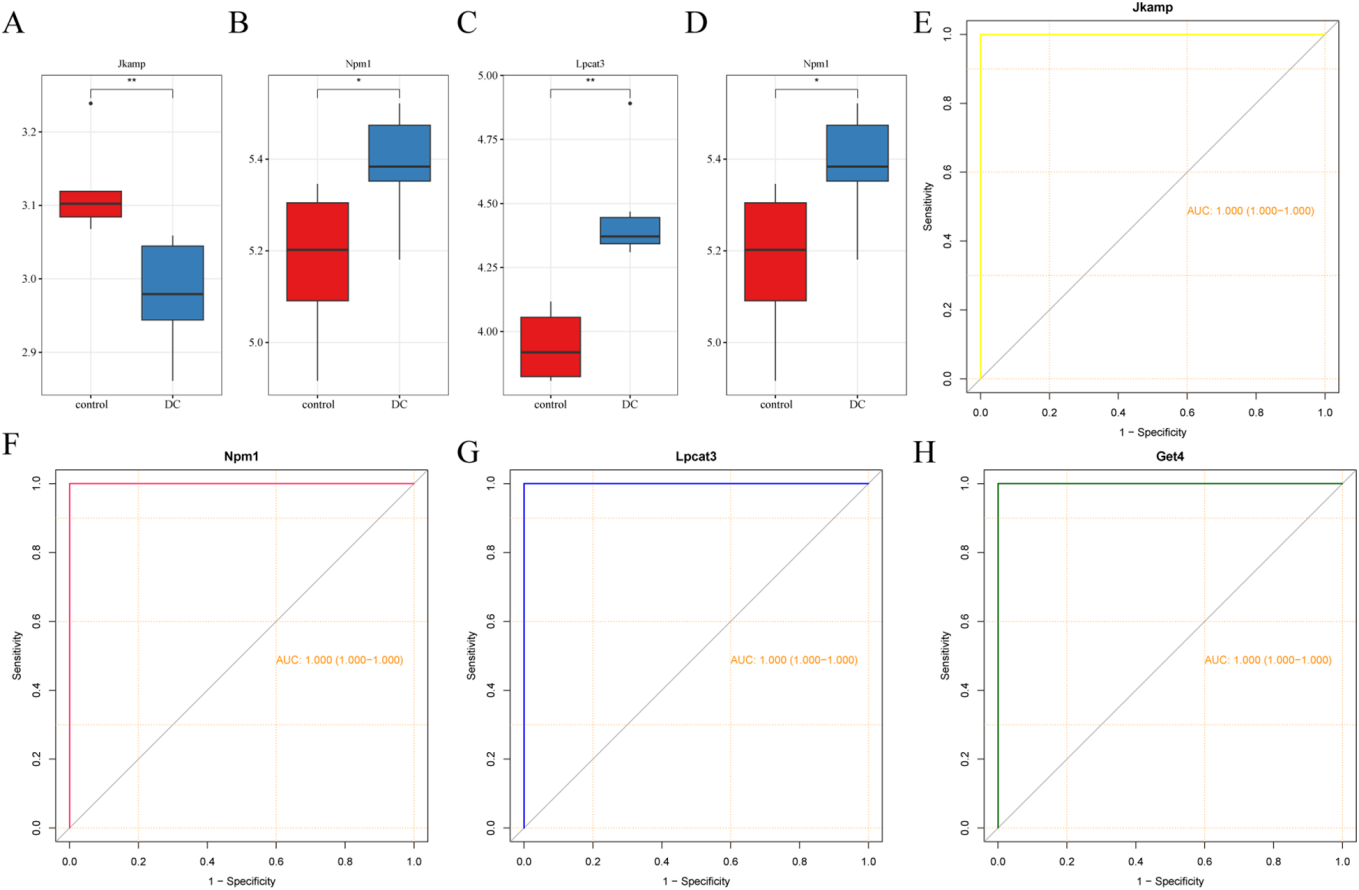
Validation of the expression and diagnostic ability of the signature genes. **(A-D)** Box plots showing the expression levels of the four signature genes. **(E-H)** ROC curves of the four signature genes. ^*^P < 0.05, ^**^ *P* < 0.01.

In the validation dataset, the expression trends of the four signature genes were consistent with those in the training set (**Figures 5A–D**). All four ROC-AUCs in the validation dataset were greater than 0.75 (**Figures 5E–H**). Overall, these results suggest that ERS-related signature genes have a predictive ability in the diagnosis of DC.

### Validation of four signature genes in a mouse model of type 1 DC

**Figure 6:**
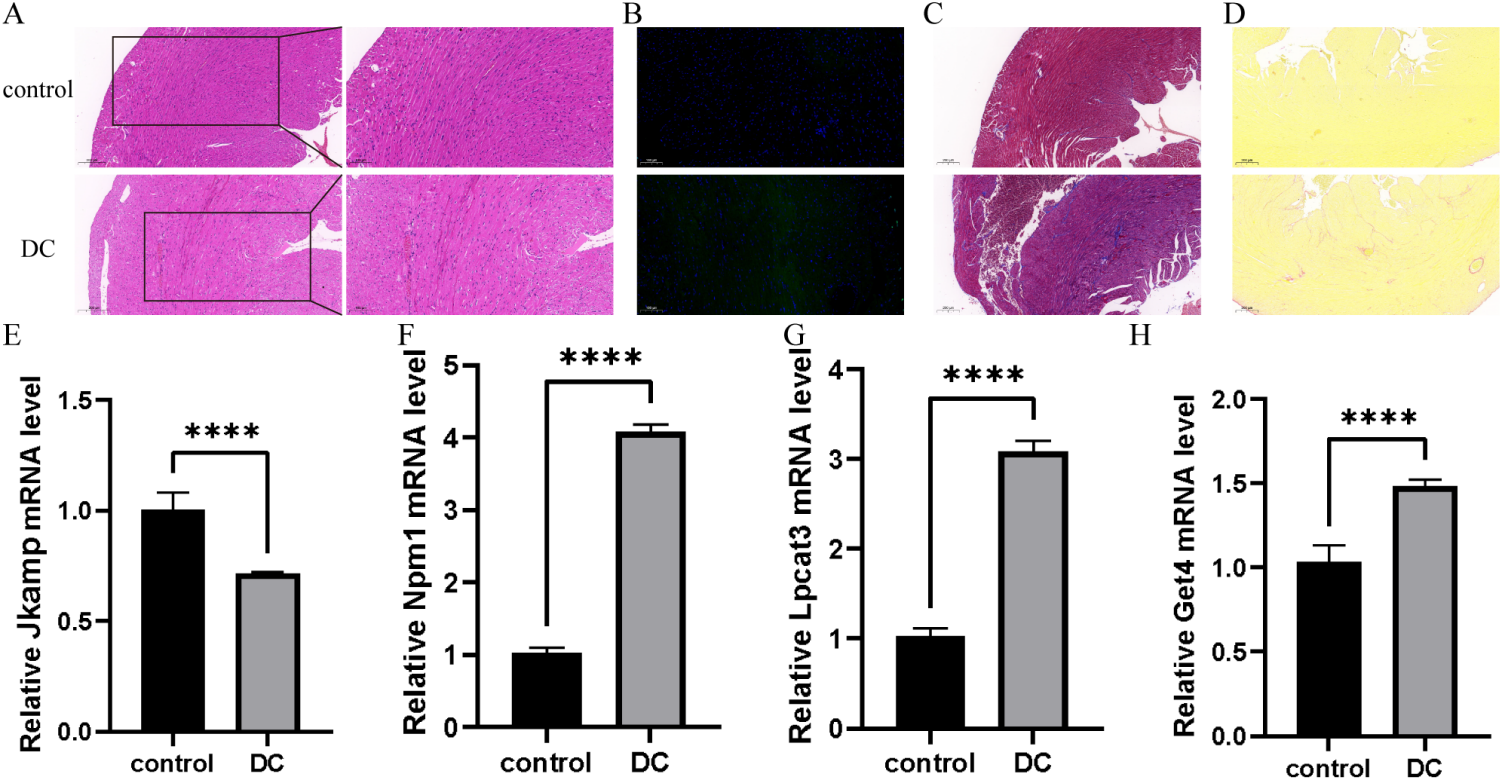
Validation of signature genes in a mouse model of type 1 DC. **(A)** Representative HE staining images to assess myocardial injury. The scale bar represents 200 and 100 μm.(n=3) **(B)** Representative Tunel staining to assess apoptosis. The scale bar represents 100 μm. (n=3) **(C, D)** Representative Masson staining images and Sirius red staining images to assess collagen fiber area. The scale bar represents 200 μm. (n=3) **(E-H)** Relative expression levels of mRNA of four signature genes in cardiac tissues by RT-qPCR. The data was presented as mean ± SD, ^****^P < 0.0001, n = 6.

As shown in **Figure 6A**, the myocardium was significantly damaged in the DC group compared with the control group, as evidenced by disorganized cardiomyocyte arrangement, edema, and inflammatory cell infiltration. As shown in **Figure 6B**, there were more apoptotic cells in the DC group compared with the control group. As shown in **Figures 6C** and **D**, there were obvious fibrotic areas in the cardiac tissue in the DC group compared with the control group. As shown in **Figures 6E–H**, RT-qPCR was employed to verify the expression levels of the four signature genes in cardiac tissues, which was consistent with the results of bioinformatics analysis.

## Discussion

Diabetic cardiomyopathy represents a significant complication of diabetes, with affected patients being 2 to 4 times more likely to develop heart failure or succumb to mortality compared to the general population (23, 32). Given that diabetes is recognized as the most prevalent disease globally, alongside the aging population and shifts in contemporary dietary patterns, the incidence of diabetic cardiomyopathy has shown a concerning upward trend (7, 23, 24, 33, 34). Consequently, it is imperative to investigate early diagnosis and prevention strategies for this condition. In this study, DC samples and ERGs obtained from public datasets identified 65 ERDEGs, from which four signature genes of type 1 DC, including Jkamp, Npm1, Lpcat3, and Get4, were identified by LASSO and Random Forest analyses. Four characteristic genes of diagnostic significance have been discovered to be closely associated with immune cells. Although the four genes we screened currently lack relevant literature confirming directly their relationship with DC, they are significant in the progression or repair of various complications associated with diabetes and cardiovascular disease. This indicates their considerable research potential in the context of DC progression. We will discuss each gene in detail below.

JNK1/mapk8-associated membrane protein (JKAMP/JAMP) is a seven-transmembrane protein situated in the cytoplasmic membrane. It interacts with JNK1 via its C-terminal domain, enhancing and prolonging JNK1 activity, which in turn increases the incidence of JNK-dependent apoptosis (35). This apoptotic process has been established as a critical factor in DC and is recognized as a consequence of ERS (36–39). Furthermore, studies examining other systemic complications of diabetes, such as diabetic osteoporosis, have confirmed that the overexpression of JKAMP appears to mitigate the adverse effects of hyperglycemia through the activation of the Wnt signaling pathway (40). These phenomena suggest that JKAMP may influence the progression of DC by modulating ERS. Nucleophosmin 1 (NPM1) is a multifunctional nucleophosmin with shuttling properties involved in a variety of cellular functions, including participation in liquid-liquid phase separation, ribosome biogenesis, and histone chaperoning and transcriptional regulation (41). Although a direct link between NPM1 and DC has not yet been established, existing evidence suggests that NPM1 can significantly influence macrophage polarization, thereby impacting the repair processes of cardiomyocytes (42). During the progression of DC, which is characterized as a typical metabolic and immune-related cardiovascular disease, the polarization of macrophages by NPM1 may represent a critical mechanism (43). LPCAT3, a gene widely expressed in key metabolic tissues such as the liver, small intestine, skeletal muscle, macrophages, and adipocytes, has been shown to directly enhance the activation of IRE1α and PERK by altering the phospholipid composition of the endoplasmic reticulum membrane, thereby triggering ERS (44, 45). These processes are regarded as potential targets for the treatment of metabolic diseases (46, 47). Interestingly, several studies have indicated that lpcat3 can significantly influence the expression levels of GPX4, thereby regulating the extent of ferroptosis and ultimately impacting the progression of DC (48, 49). Golgi to ER traffic protein 4 (GET4) and Get5 form a complex that competitively binds ribosomes with SRP and directs tail-anchored proteolytic delivery to the endoplasmic reticulum (50). Mutants with GET dysfunction are more susceptible to ERS (51). DC, whether its rate of progression is believed to be significantly influenced by ERS (36–39) or regarded as a classic metabolic disease (23, 43), appears to be inextricably linked to the four aforementioned molecules. Unfortunately, there are currently no definitive studies confirming the relationship between these four molecules and DC; however, this also suggests the research potential of these molecules.

The present study further analyzed immune cell infiltration and revealed that there were significantly more B cells naive in the DC group than in the control group, so it was hypothesized that DC was associated with abnormalities in the immune system. Inhibited activation of the NF-κB signaling pathway in activated B cells decelerated the progression of type 1 DC (52). In addition, this study demonstrated the correlation of Npm1 and Get4 with immune cells, suggesting that they may influence the development of DC by affecting immune cells. Therefore, ameliorating the abnormal immune status may also be a promising therapeutic strategy for DC.

Numerous prior studies on DC have sought to identify potential biomarkers and therapeutic targets for intervention. These investigations frequently emphasize aspects such as immune metabolism, with some specifically addressing the regulation of the immune microenvironment (53–56). ERS is the earliest pathophysiological process to be fully activated in DC (36–38, 57). Molecular markers derived from related molecules appear to possess superior properties for early diagnosis. Furthermore, ERS and the consequent apoptosis of cardiomyocytes represent critical factors influencing the progression rate of DC (36–38, 57). Consequently, the molecular markers identified from these related molecules are more effective as therapeutic targets. However, some existing studies utilize high-throughput technical methods, such as metabolomics and liquid chromatography-mass spectrometry (LC-MS), which appear to play a more significant role in identifying key pathways and molecules involved in DC (58).

Additionally, there are some limitations of this study. Although some studies have indicated that mice are the only mammals that can provide such a rich resource of genetic diversity while also allowing for extensive genome manipulation, making them a powerful tool for modeling human cardiovascular diseases, particularly diabetic cardiomyopathy (23, 59–61). In these models, the trends of various physiological indicators in mice, such as left ventricular contractility and left ventricular ejection fraction, align with those observed in humans, establishing the mouse diabetic cardiomyopathy model as a primary technology for studying this disease (23, 60, 62). However, it is important to note that numerous studies have highlighted significant deviations in conclusions derived from mouse models when translated to clinical and technical applications (23, 63). Furthermore, intervention methods used in humans cannot be fully replicated in diabetic mice (23, 62). Therefore, our study requires further supplementation and validation through clinically relevant research in the future. In addition to this limitation, we also face several shortcomings, including the need for further investigation into the expression and diagnostic value of these four genes at the protein level, as well as the absence of additional experiments to validate the effects of these characteristic genes on immune cells and heart function.

## Methods

### Data collection

**Figure 7:**
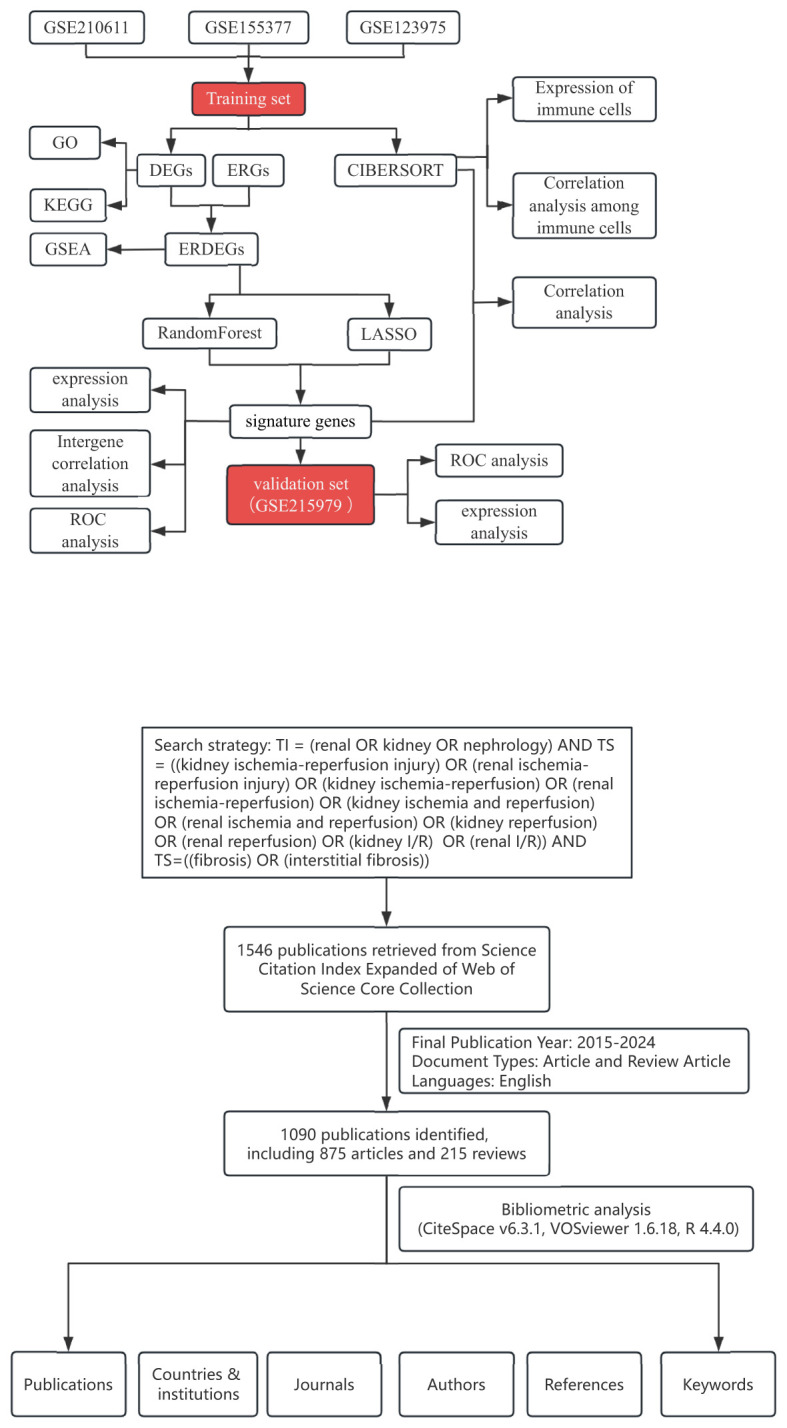
Workflow diagram of this study. The training set acquires ERS-related signature genes and performs immune infiltration analysis. In addition, the validation set judges the diagnostic performance of the signature genes.

The Gene Ontology (GO) knowledgebase (http://geneontology.org/) was searched to collect ERGs, including regulation of response to endoplasmic reticulum stress (GO:1905897), unfolded protein binding (GO:0051082), response to endoplasmic reticulum stress (GO:0034976), and endoplasmic reticulum unfolded protein response (GO:0030968), and 345 ERGs were obtained after removing duplicates. Gene expression profiling of type 1 DC was obtained from the GEO (http://www.ncbi.nlm.nih.gov/geo) database, including GSE210611, GSE155377, GSE123975, and GSE215979, working according to the flowchart in **Figure 7**. Among them, GSE210611 (3 CON and 3 DC), GSE155377 (4 CON and 4 DC), and GSE123975 (4 CON and 4 DC) were constructed as a multichip dataset containing 13 CON and 13 DC by R (version 4.3.1) software, and the remove batch effect function of the limma (version 3.60.2) package could be applied to remove batch effects. Principal Component Analysis (PCA) was applied to assess whether the batch effect had been removed. GSE215979 (3 CON and 3 DC) was set as the external validation dataset.

### Identification of differentially expressed genes

DEGs were analyzed between the DC and control groups using the R package “limma” (version 3.60.2) for the multi-chip dataset. Genes filtered according to the threshold |log_2_fold change(FC)|> 0 and *P* < 0.05 were selected as DEGs, and volcano plots were generated to visualize the results. In addition, gene set enrichment analysis was utilized to identify the most significant functional terms between the DC and control groups (64).

### Screening and functional enrichment analysis of ERDEGs

Draw Venn Diagram (https://bioinformatics.psb.ugent.be/webtools/Venn/) intersected ERGs with DEGs in the multichip dataset to obtain ERDEGs, whose gene expression was demonstrated by heatmaps created with the “pheatmap” package (version 1.0.12). To further elucidate the biological functions of ERDEGs, GO enrichment analysis and KEGG pathway analysis were performed via “ClusterProfiler” (version 4.12.0), whose results were plotted using the “ggplot2” package (version 3.5.1). Screening for BP, CC, MF, and KEGG pathways with *P <*0.05 (65, 66).

### Identification and ROC analysis of ERS-related signature genes by LASSO algorithm

The R package “glmnet” (version 4.1.8) was applied to obtain ERS-related signature genes by the LASSO algorithm with 10-fold cross-validation to determine the optimal value of the penalty parameter λ. LASSO algorithms are often interpreted by cross-validation graphs and regression coefficient path graphs. First, the cross-validation curve is applied to select the optimal lambda value. The X-axis is the logλ of the penalty coefficient, and the Y-axis is the likelihood deviation. The smaller the Y-axis is, the better the fitting effect of the equation is. The top number is the number of variables left in the equation for different λ. There are usually two dotted lines, the lambda with the smallest deviation but the highest model fit (lambda. min) on the left, and the lambda value with the smallest deviation but the more concise model (lambda.1se) on the right. Based on the best prediction performance and feature selection capability, lambda. min is selected in this paper. Second, regression coefficient path graph, each line in the graph represents a variable, the ordinate is the coefficient, and the upper abscissa is the number of non-zero coefficients in the model under different regularization parameters. The lower horizontal coordinate is the normalized normalized parameter. The figure shows the variation trajectories of variable coefficients under different regularization parameters. When the regularization is larger, the complexity of the model is lower, so most parameters will approach 0. We can know which features contribute more or less to the prediction of the model through this figure, and we can initially use the features with greater contributions in subsequent analysis.

Random Forest is an integrated learning method based on decision trees, where multiple decision trees are constructed and their predictions are aggregated for classification or regression tasks to improve overall accuracy and stability (67). One of the most important results of Random Forests in machine learning is the assessment of significance based on the Gini index and the final identification of the featured genes, which can be done with the “RandomForest” (version 4.7.1.1) package of the R software and visualized by drawing dotplots with varImpPlot. The crossover genes of LASSO and Random Forest were then identified as the optimal ERS-associated signature genes for the next step of the study.

ROC analysis was performed to determine the diagnostic validity of the four signature genes with the “pROC” package (version 1.18.5). ROC curve is a curve obtained by plotting the true positive rate and false positive rate, which can reflect the relationship between sensitivity and specificity. The horizontal axis represents the false positive rate (1- specificity) and the vertical axis represents the true positive rate (sensitivity). ROC-AUC reflects the value of diagnostic tests. The larger the area, the closer to 1.0, the higher the diagnostic authenticity. The closer it is to 0.5, the lower the accuracy of the diagnosis. When it is equal to 0.5, it has no diagnostic value. ROC-AUC was applied to estimate the diagnostic ability to differentiate DC from the control group.

### Immune cell infiltration analysis

Immune cell subtypes in the multichip dataset were evaluated by the CIBERSORT algorithm with the LM22 gene feature matrix, and values with *P* < 0.05 were considered statistically different (68). Correlations between immune cells and between immune cells and ERS-related signature genes were analyzed using Spearson analysis.

### Construction of a mouse model of type 1 DC

C57BL/6j male mice (20-25 g) were raised in the standard barrier environment of the Animal Experiment Center at Renmin Hospital of Wuhan University, maintained at a temperature of 21-24°C and a humidity of 50-60%, with a light and dark cycle of 12 hours. This experiment received approval from the Wuhan University Committee on Animal Care and Utilization (IACUC Issue No. 20230805C). Upon reaching 6 to 8 weeks of age, the mice were randomly assigned to either a control group or a diabetes group, with a minimum of six mice in each group. The diabetic group received intraperitoneal injections of streptozotocin (STZ, Boagang, China) dissolved in a citric acid buffer (pH 4.2-4.5) at a dose of 50 mg/kg for five consecutive days. In contrast, the control group was administered an equal volume of STZ-free citrate buffer. Fasting blood glucose levels were measured on days three and seven following the injections; mice exhibiting fasting blood glucose levels of 11.1 mM or higher were classified as diabetic. Subsequently, the mice were maintained on a continuous feeding regimen for 12 weeks, during which blood glucose levels were assessed every four weeks to confirm that the blood glucose levels of the diabetic mice remained consistently above 11.1 mM. At the conclusion of the 12th week, the mice were euthanized in accordance with ethical guidelines, and their hearts were excised. Some hearts were designated for immediate mRNA extraction, while others were fixed in 4% paraformaldehyde for subsequent pathological analysis. This allocation was performed randomly. Mice that did not develop the disease model were euthanized following the protocols outlined in the ethics manual.

### Cardiac histomorphometry

The fixed heart tissues were embedded in paraffin and cut into 4 μm thick sections. They were then stained with hematoxylin and eosin (HE), Tunel, Masson, and Sirius red which are shown below.

### Hematoxylin-eosin

The paraffin sections were dewaxed in a series of solutions: Dewaxing Clear Solution I for 20 minutes, Dewaxing Clear Solution II for 20 minutes, Absolute Ethanol I for 5 minutes, Absolute Ethanol II for 5 minutes, and 75% Alcohol for 5 minutes. Subsequently, the sections were immersed in a hematoxylin staining solution for 5 minutes, followed by a wash with double-distilled water. The sections were then differentiated using hematoxylin differentiation solution for a few seconds, rinsed with double-distilled water, and treated with hematoxylin blue-returning solution to restore the blue color, followed by another rinse with double-distilled water. Next, the sections were dehydrated in 85% Alcohol and 95% Alcohol for 5 minutes each, stained with eosin stain for 5 minutes, and then dehydrated and clarified in absolute ethanol and dewaxing clear solution. Finally, the sections were sealed with neutral gum. The structure and morphology of the myocardial fibers were observed using optical microscopes.

### Terminal deoxynucleotidyl transferase-mediated dUTP nick-end labeling

Terminal deoxynucleotidyl transferase-mediated dUTP nick-end labeling (TUNEL) was used to examine the myocardial cell apoptosis with an *in situ* cell death detection kit (Nanjing Jiancheng Bioengineering Institute, China) according to the manufacturer’s instructions. Briefly, the heart tissues of each group were embedded in paraffin and cut into 5 μm thick sections. Then the sections were stained with TUNEL reaction mixture for 60 min and immersed into 4’,6-diamidino2-phenylindole (DAPI) to stain nuclei for 30 min. Apoptotic cells were observed under a light microscope with an excitation wavelength of 585–600 nm.

### Masson’s trichrome stain

After dewaxing the paraffin sections to water using the aforementioned method, perform the following steps in sequence: (1) Immerse in potassium dichromate (Servicebio, G3326, China) overnight; (2) Stain with iron hematoxylin (Servicebio, G3326, China) for 10 minutes, followed by thorough washing with water; (3) Return to blue using Masson blue solution (Servicebio, G3326, China) for 5 minutes, and wash well with water; (4) Stain with Ponceau magenta (Servicebio, G3326, China) for 10 minutes; (5) Wash with phosphomolybdic acid solution (Servicebio, G3326, China) for 2 minutes; (6) Wash with weak acid working solution for 1 minute; (7) Stain with aniline blue dyeing solution (Servicebio, G3326, China) for 1 minute; (8) Wash with weak acid working solution for 1 minute; (9) Sequentially immerse in 95% alcohol, 100% alcohol, and xylene to dehydrate and render the sections transparent; (10) Seal the slide with neutral gum. Finally, use an optical microscope to observe the deposition of collagen fibers in the myocardium, where collagen fibers appear blue, while muscle fibers, cytoplasm, and cutin are red.

### Sirius Red

After removing the paraffin sections and dewaxing them in water, proceed with the following steps in sequence: (1) stain with Sirius scarlet for 8 minutes; (2) dehydrate using absolute ethanol for 5 minutes; (3) seal with neutral gum. Utilize a 400× optical microscope to observe the deposition of collagen fibers in the myocardium, where collagen fibers appear red and other tissue components are displayed in yellow.

### Real-time fluorescence quantitative PCR

Total RNA was extracted from cardiac tissues using an RNA extraction kit (RC113-01; Vazyme, China). Reverse transcription and quantification were performed using SweScript All-in-One RT SuperMix for qPCR (One-Step gDNA Remover) (Servicebio, G3337, China) and 2×Universal Blue SYBR Green qPCR Master Mix (Servicebio, G3326, China). Finally, RT-qPCR was performed on a Lightcycler 480II Real-Time Fluorescence Quantitative PCR System (Roche, Germany). β-actin was used for expression normalization. The primers are shown in **Table 1**, and the relative expression of the genes was determined by the 2^-ΔΔCT^ method.

**Table 1:** Primes used for RT-qPCR analysis.

Gene	Forward primer	Reverse primer
Npm1	AGGACGATGATGAGGACGATGAG	CCCTTTGATCTCGGTGTTGATGG
Jkamp	CACGATGCTCTACAACCCAAGTC	CATGCGATCTTCTTCACCAGGAG
Get4	CCGAGGCTTCCGAAGTGAGG	CAGCAGCAGAAACCAGATGAAATTG
Lpcat3	CACCGTCACTGCCGTTATTACTAC	TCCCGTCTTTGCCTCCATCG
β-actin	GTGACGTTGACATCCGTAAAGA	GTAACAGTCCGCCTAGAAGCAC

### Statistical analysis

All statistical analyses were performed by R software (version 4.3.1). Student’s t-test or one-way ANOVA was applied to assess differences between the two groups. *P* < 0.05 indicated statistical significance.

## Conclusion

In summary, four signature genes (Npm1, Jkamp, Get4, and Lpcat3) have been tentatively identified as potential diagnostic markers of type 1 DC, which may be influenced by controlling ERS and immune cells. The results of this study provide new insights into the development of new targets for the diagnosis and treatment of DC.

## Data Availability

The datasets presented in this study can be found in online repositories. The names of the repository/repositories and accession number(s) can be found in the article/supplementary material.
